# Relationship between serum endothelin-1 and in-stent restenosis following vertebral artery stenting

**DOI:** 10.1007/s10072-023-07276-9

**Published:** 2023-12-29

**Authors:** Fang He, Changyang Zhong, Chunli Wu, Yuan Liu, Shufeng Yu

**Affiliations:** 1 Physical Examination Center, Hangzhou Third People’s Hospital, Hangzhou, China; 2 Cerebrovascular Disease Department, Hangzhou Third People’s Hospital, Hangzhou, China; 3 Department of Radiology, Zhejiang People’s Hospital, Hangzhou, China

**Keywords:** Serum endothelin-1, Vertebral artery, Stents, Restenosis

## Abstract

The study objective was to investigate the relations between serum endothelin-1 and in-stent restenosis in vertebral artery stenting. Sixty-eight patients undergoing re-examination of vertebral artery stenting in the Department of Cerebrovascular Disease, Hangzhou Third People’s Hospital, between April 2019 and October 2022, were invited to participate. According to the presence of vertebral artery stenting, patients were divided into the restenosis (*n* = 19) or non-restenosis (*n* = 49) groups. General clinical data and endothelin-1 levels were compared between the groups. Logistic regression analysis was used to explore the relations between endothelin-1 level and risk for in-stent restenosis. Receiver operating characteristic curves were drawn to test the diagnostic value of serum endothelin-1 level for in-stent restenosis. Compared with the non-restenosis group, restenosis group levels of low-density lipoprotein, triglycerides, and endothelin-1 were significantly higher (*p* < 0.05) Multivariate logistic regression analysis showed that endothelin-1, stent length, and low-density lipoprotein were independently associated with in-stent restenosis (odds ratio = 1.502, 95% confidence interval: 0.042 ~ 0.212, *p* = 0.000; odds ratio = 1.899, 95% confidence interval: 1.116 ~ 2.237, *p* = 0.000; odds ratio = 1.899, 95% confidence interval: 1.228 ~ 3.337, *p* = 0.001, respectively). Area under the curve for serum endothelin-1 in the diagnosis of vertebral artery in-stent restenosis was 0.938. The best diagnostic cut-off value was 11.94 ng/L. Sensitivity was 89.5%. Specificity was 85.7%. These cumulative data indicate that endothelin-1 level is independently associated with in-stent restenosis.

## Introduction

Stroke has become a serious threat to human health, due to its high incidence and disability rates, and China ranks first globally in both stroke incidence and mortality [1]. Approximately 25% of strokes occur in the vertebrobasilar artery system, and vertebral artery stenosis is an important cause of ischemic cerebrovascular disease [2]. Furthermore, New England Medical Center data show that 20% of posterior circulation strokes are caused by lesions at the beginning of the vertebral artery [3]. Current treatments for vertebral artery stenosis include drug therapy, surgery, percutaneous transluminal angioplasty, and stent angioplasty.

Among available interventions, endovascular stent implantation has become among the most effective treatments for patients with vertebral artery stenosis [4]. The Vertebral Artery Ischemic Stenting Trial reported that medical treatment is favored for intracranial vertebral stenosis because of the higher operative risk. In contrast, stent implantation for extracranial symptomatic vertebral stenosis has been acknowledged as a safe option for reducing long-term stroke risk [5]. However, the restenosis rate after vertebral artery stenting is the highest among all intracranial and extracranial vascular stenting procedures [6]. A multicenter prospective studyreported a restenosis rate as high as 35% after stenting of the extracranial vertebral artery [7]. The stenosis can affect cerebral hemodynamics, leading to stroke recurrence. Therefore, early prediction and treatment of in-stent restenosis (ISR) following surgery can improve patient prognosis.

Neointimal hyperplasia is the basis for the pathophysiological development of ISR, which is triggered by proinflammatory molecules released by endothelial injury. Inflammatory molecules play a key role in the stages of stent thrombosis and proliferation [8]. Serum endothelin-1 (ET-1) is an important protein molecule, the expression levels of which reflect vascular endothelial cell injury and inflammation [9]. However, neither changes in serum ET-1 levels before and after vertebral artery stenting, nor its effectiveness for predicting ISR risk after this stenting, have been studied. Therefore, this postoperative early prediction study explored the predictive value of serum ET-1 changes for risk of ISR after vertebral artery stenting, toward determining whether this can serve as an effective biomarker for preventative and mechanistic ISR research after vertebral artery stenting.

## Materials and methods

### Patients and eligibility criteria

All participants provided verbal and written informed consent before study enrollment. This study conformed to the single center, observational,cohort study, the ethics of the study complied with the Declaration of Helsinki, and was approved by the Biomedical Ethics Committee of Hangzhou Third People’s Hospital (Scientific Research Medical Ethics (021)-7.19). Participants were all inpatients at the Department of Cerebrovascular Diseases of the Third People’s Hospital of Hangzhou between April 2019 and October 2022. Sixty-eight patients (45 males and 23 females) who met the surgical indications for vertebral artery stenting and eligibility criteria were invited to participate.

#### Inclusion criteria

Inclusion criteria were: 1) Age 18–80 years; 2) Relevant vascular examination suggested that the offending vessel was located in the vertebral artery, the symptomatic stenosis rate of the extracranial vertebral artery was ≥ 50% or asymptomatic stenosis rate was ≥ 70%, the symptomatic stenosis rate of the intracranial vertebral artery was ≥ 70%, Standard antiplatelet therapy (e.g., bay aspirin 100 mg or clopidogrel 75 mg, etc.) and statin therapy (e.g., atorvastatin 20 mg, etc.) was ineffective; 3) Digital subtraction angiography (DSA) determined that the lesion length could be covered by a single stent and the reference vessel diameter was ≥ 2 mm; 4) Informed consent was obtained.

All patients underwent head and neck computed tomography (CT) angiography (CTA), DSA, head MRI, or CT after 12 months. The number of ischemic events related to the stenosis vessel (e.g., transient ischemic attack including vertigo and unsteady gait, new cerebral infarctions) within 12 months was recorded. If symptoms occurred, DSA or CTA of the head and neck vessels was immediately reexamined to observe vertebral artery stent morphology.

#### Exclusion criteria

The exclusion criteria were: 1) Severe bleeding, intracranial or extracranial bleeding, or active gastrointestinal ulcer within the past 3 months; 2) Drug intolerance (e.g., contrast media, aspirin, clopidogrel); 3) Poor general patient health (e.g., uncontrolled malignant hypertension, serious medical diseases, malignant tumor); and 4) Poor blood vessel conditions (e.g., vascular distortion, variation, severe stenosis).

### Treatment methods

All patients received dual antiplatelet therapy with aspirin (100 mg/d) and clopidogrel (75 mg/d) for more than 3 days before surgery. The procedure was performed under local anesthesia by a senior neurologist. A 6-French sheath was placed in the femoral artery and systemic heparinization with an activated clotting time of 2–3 times the baseline was maintained during the procedure. Then, a 6-French guiding catheter (Cordis, Miami Lakes, FL, USA) was placed in the subclavian artery over a 0.035 inch guide wire. Next, stenotic lesions were crossed with 0.014 inch guidewires (Cordis) under road-map fluoroscopy. The appropriate stent was subsequently used for the lesion. The diameter of the deployed stent was based on the diameter of the distal normal vessel and the length of the stent had to cover 2 mm of normal lumen on either side of the lesion. Balloon expandable bare-metal stents (Herculink, Abbott Vascular, USA) were deployed as the first choice. The Apollo Stent (MicroPort Medical, Shanghai, China) were used as intracranial arterial system stents only when Herculink implantation was predicted to be challenging due to the tortuosity of the proximal vertebral artery [10]. Finally, angiography was performed to evaluate the residual stenosis. Dual antiplatelet drugs (aspirin and clopidogrel) were continued for at least 6 months, after which the patient was switched to life-long aspirin therapy. All patients were given a standardized prevention program, including smoking cessation and management of blood pressure, blood glucose, and serum lipids.

Postoperative restenosis was defined as: 1) A 50% stenosis of the extracranial vertebral artery within 5 mm of the stent or 2) A was 50% stenosis within 5 mm of the intracranial vertebral artery stent, or the absolute diameter of the vessel was reduced by 20% upon review. The rate of extracranial stenosis was measured according to the American Symptomatic Carotid Endarterectomy Trial criteria. The rate of intracranial stenosis was measured using the Warfarin–Aspirin Symptomatic Intracranial Disease method. According to these criteria, patients were divided into the non-restenosis or restenosis groups.

### Serum ET-1 and biochemical indicators

Blood samples were collected from all patients before surgery (i.e., within 24 h after admission). Serum ET-1 was detected by a double-antibody two-step sandwich enzyme-linked immunosorbent assay. Samples or standards, biotinylated anti-ET-1, and horseradish peroxidase-labeled avidin were added to the microwells coated with anti-ET-1 antibody in turn. After thorough washing, the microwells were stained with substrate TMB, which turned blue under the catalytic activity of peroxidase. Finally, acid is used to turn the solution yellow in the presence of ET-1, with a positive correlation between the depth of color and ET-1 in the sample. Absorbance (optical density [OD] value) was measured with a microplate reader at 450 nm wavelength, and the sample concentration was calculated.

Diluted serum samples (40 μl), ET-1 antibody (10 μl), and horseradish peroxidase labeled streptavidin (50 μl) were added to a 96-well plate and incubated for 60 min at 37 °C with gentle shaking. After five washes, the chromogenic reagent (50 μl) was added and incubated at 37 °C for 10 min. Finally, the termination solution was added. The OD values at 450 nm wavelength were measured using an ultraviolet spectrophotometer. The concentration standard curve was drawn, and the linear regression equation was calculated based on OD value and standard concentration. The concentration of each sample was then calculated. The kit was purchased from Shanghai Hengyuan Biotechnology Co., Ltd.

### Clinical data

Detailed medical history, medication history, and physical examinations were collected for all participants. Clinical data included age, sex, smoking history, drinking history, and blood pressure.

### Statistical analysis

Statistical analyses were performed using SPSS version 21.0 (International Business Machines Corp., Armonk, NY, USA). Patient characteristics are described as frequency (percent) or mean ± standard deviation (SD). Significant between-groups differences were assessed by one-way analysis of variance. The chi-squared test was used to analyze categorical data. Diagnostic efficiency was assessed by receiver operating characteristic (ROC) curve analysis. Multivariate logistic regression model was used to analyze risk factors. For all analyses, *p* < 0.05 was considered statistically significant. Figures were drawn using SPSS (International Business Machines Corp.) and GraphPad Prism version 8.0 (GraphPad Software, San Diego, CA, USA) software programs.

## Results

Sixty-eight patients (age range, 42–82 years), each of whom had undergone a single stent implantation, were included in analyses. ISR prediction analysis (Table 1) showed no significant between-group differences in age, gender, high blood pressure, drinking, smoking, creatinine, high-density lipoprotein cholesterol, triglycerides (TG), or uric acid levels (all, *p* > 0.05). Compared with the non-restenosis group, LDL, TG, and ET-1 levels in the restenosis group were significantly higher (*p* < 0.05, *p* < 0.01, Table 1).

**Table 1:** Detailed clinical information for vertebral artery stenting and healthy volunteer groups (mean ± SD)

Characteristic	Restenosis group (*n* = 19)	No restenosis group (*n* = 49)	t-value	*P*-value
Gender			0.007	0.392
Male	12	33		
Female	7	16		
Age	65.84 ± 7.93	68.84 ± 7.95	1.393	0.168
Hypertension	9	29	0.775	0.379
Smoking	10	21	0.576	0.412
Diabetes	12	30	0.022	0.883
Coronary heart disease	1	18	1.074	0.427
HDL	1.44 ± 0.40	1.34 ± 0.23	1.292	0.291
TC (mmol/L)	4.52 ± 1.21	4.69 ± 0.69	1.585	0.376
IL10 (pg/ mL)	4.03 ± 1.14	4.12 ± 1.22	0.156	0.938
TG (mmol/L)	5.88 ± 0.68	4.08 ± 0.87	8.091	0.000
Glycosylated hemoglobin	8.40 ± 2.43	8.52 ± 2.66	0.166	0.869
LDL (mmol/L)	4.16 ± 0.69	2.27 ± 0.66	10.429	0.000
ET-1	43.33 ± 7.51	9.81 ± 1.09	8.340	0.000

There were no significant between-groups differences in age, gender, hypertension, or other indicators (*p* > 0.05). Compared with the non-restenosis group, the levels of low-density lipoprotein (LDL), TG and ET-1 in the restenosis group were significantly higher (*p* < 0.05 and *p* < 0.01, respectively)

### Surgical data

Neither lesion location nor stent placement time differed between the groups (both, *p* > 0.05). Compared with the non-restenosis group, the restenosis stent diameter and stent length ratio were significantly higher in the restenosis group (both, *p* < 0.01, Table 2).

**Table 2:** Surgical data for the vertebral artery stenting and healthy volunteer groups

Characteristic	Restenosis group (*n* = 18)	No restenosis group (*n* = 50)	*P*-value
Stent diameter (mm) M (Q1,Q3)	3.5 (3.0,4.0)	4.5 (4.0,4.5)	0.032
Bracket length (mm) M (Q1,Q3)	15 (13.0,17)	11 (9.0,15.0)	0.001
Timing of stent implantation M, year (Q1,Q3)	0.8 (0.6,1.4)	0.7 (0.5,1.0)	0.081
Location of stenting			
LVAO	9	28	0.892
RVAO	10	22	0.904

Compared with the non-restenosis group, the restenosis stent diameter and stent length ratio were significantly increased (*p* < 0.01)

Pearson correlation analysis showed that when comparing the relevant indices between the two groups, ET-1, LDL, TG, stent diameter, and stent length were positively correlated with ISR (all, *p* < 0.05). Diabetes, smoking, and fasting blood glucose were not significantly associated with ISR (all *p* > 0.05, Table 3).

**Table 3:** Correlation analysis

Characters	*r* value	*P* value
Stent diameter (mm)	0.493	< 0.001
Stent length	0.746	< 0.001
LDL	0.789	< 0.001
TG	0.706	< 0.001
ET-1	0.716	< 0.001

Comparing the relevant indices between-groups, ET-1, LDL, TG, stent diameter, and stent length were positively correlated with ISR (*p* < 0.05)

Multivariate logistic regression analysis showed that ET-1, stent length, and LDL were independently associated with ISR (all *p* < 0.05, Table 4).

**Table 4:** Multivariate logistic regression analysis

Characters	*β* value	*P*-value	Wald	OR value	95% CI
Stent diameter (mm)	0.061	0.494	3.845	2.067	0.032–1.102
Stent length (mm)	0.067	0.000	0.688	1.899	1.116–2.237
LDL	− 0.232	0.001	4.442	0.768	1.228–3.337
TG	0.051	0.290	1.068	0.882	0.147–0.450
ET-1	0.007	0.000	4.934	1.502	0.042–0.212

Multivariate logistic regression analysis showed that ET-1, stent length, and LDL were independently associated with ISR (all *p* < 0.05)

**Fig. 1:**
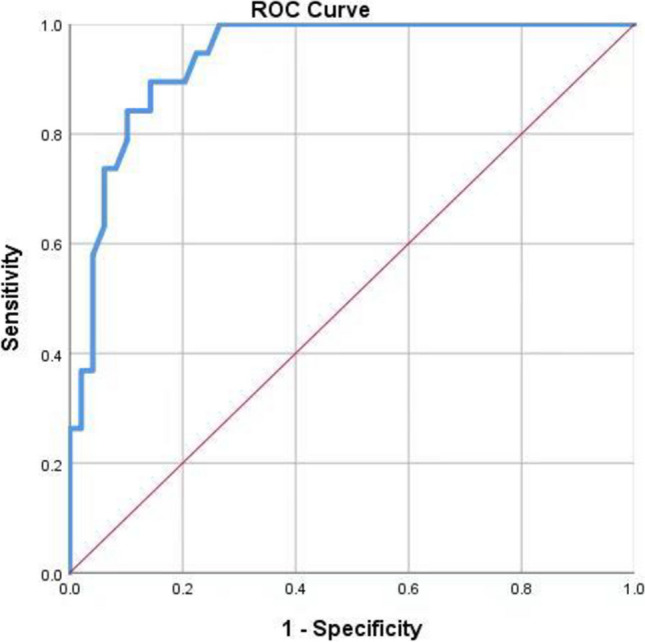
Predictive value of ET-1 in coronary in-stent restenosis value. The area under the curve of serum ET-1 in diagnosis of vertebral artery ISR was 0.938, best diagnostic cut-off value was 11.94 ng/L, sensitivity was 89.5%, and specificity was 85.7%

Area under the curve of serum ET-1 for diagnosis of vertebral artery ISR was 0.938. The best diagnostic cut-off value was 11.94 ng/L. Sensitivity was 89.5%. Specificity was 85.7% (Fig. 1).

## Discussion

Abnormal proliferation and migration of vascular endothelial cells is the main cause of ISR. Vascular endothelial cells provide a barrier between the vascular system and the underlying tissue, and mediate vascular relaxation and contraction by secreting nitric oxide (NO), ET-1, angiotensin, and growth factors [11]. Atherosclerosis may inhibit the proliferation and migration of vascular endothelial cells by inducing the secretion of heparin-like substances, smooth muscle relaxant factor, and smooth muscle contractile factor [12–14]. Vascular endothelial injury may occur in patients with peripheral arterial occlusive disease after interventional treatment, reducing secretion of factors that inhibit the proliferation and migration of smooth muscle cells, which may lead to ISR [15]. Damaged vascular endothelial cells may further promote thrombosis and vascular constriction, leading to ISR [16]. Therefore, our goal was to evaluate the damage to vascular endothelial cells following endovascular treatment, which may have important clinical value for predicting ISR occurrence.

ET-1 is a vasoconstrictor secreted mainly by endothelial cells. It activates Ca^2+^ channels in vascular smooth muscle cells by binding to receptors, resulting in the contraction of vascular smooth muscle cells [17]. Under normal circumstances, there is a dynamic balance between vasoconstrictor factors (e.g., ET-1) and vasodilator factors (e.g., NO). Endothelial cells produce NO, which inhibits neointimal hyperplasia that depends on vascular smooth muscle cell migration and proliferation, reduces the association of inflammatory cells with the vascular wall, and inhibits thrombus formation by reducing platelet adhesion and aggregation [18]. Herein, patient serum ET-1 levels were positively correlated with vertebral artery stent restenosis, suggesting a role in the pathological progression of vertebral artery stent restenosis. Specifically, the association suggests that higher levels of ET-1 are associated with a higher likelihood of vertebral artery stent restenosis. These data suggest that increased ET-1 secretion after vascular stent implantation disrupts this balance, leading to enhanced platelet adhesion, thrombosis, abnormal platelet proliferation and migration, and ultimately restenosis. This study confirms, for the first time, that serum ET-1 may be an independent risk factor for ISR after vertebral artery stenting in patients with vertebral artery atherosclerosis and stenosis. This finding provides a basis for understanding the disease evolution and early diagnosis of ISR in patients with vertebral artery stenosis, and provides a reference for early treatment after vertebral artery stenting. Early intervention and regular treatment for patients with elevated ET-1 may help reduce the incidence of vertebral artery restenosis, avoid secondary surgery, and improve patient quality of life.

Consistent with a previous report [19], stent diameter was a risk factor for ISR herein. Our data indicate that smaller diameter stents present a higher risk for ISR after stent placement in the vertebral artery ostium (VAO), consistent with our previous findings. Lederman et al. [20] indicated that ISR in renal artery interventions might be related to artery diameter; they found a higher ISR rate in vessels of diameter < 4.5 mm (36%) compared that in vessels > 4.5 mm (12%). We therefore presume that stenting in smaller arteries may lead to stagnation and more easily cause microscopic injuries to the segment, resulting in ISR. These cumulative data provide valuable interventional neuroradiologists insight into which patients may benefit from stenting.

Total cholesterol, smoking, and diabetes mellitus were identified as independent risk factors for ISR. Smoking is recognized as an independent risk factor for atherosclerosis; however, the relation between smoking and ISR is controversial. Previous studies have shown that smoking has a negative impact on late mortality in patients undergoing coronary artery bypass grafting. Smoking also appears to enhance the antiplatelet effects of clopidogrel [21]. In contrast, Rittersma et al. [22] reported that smoking reduces ISR risk. Ten of 31 studies analyzing the correlation between smoking and ISR concluded that smoking was not significantly associated with ISR after percutaneous coronary stent implantation. Our results are consistent with this, though varying conclusions may be due to differences in samples, including ethnicity. Zhou et al. [23] also identified diabetes as a risk factor for ISR, but not for future restenosis. This is consistent with the findings herein, that diabetes is one of several risk factors but not an independent risk factor. Previous studies [24, 25] have identified smoking, diabetes, dyslipidemia, and female sex in relation to higher rates of restenosis after carotid artery stenting. This may be due to anatomical and physiological differences between anterior and posterior circulation. However, it also suggests that patient smoking cessation, alcohol restriction, and standard blood pressure, blood glucose, and serum lipid level managements are essential.

This study was not without several limitations. Although it was a prospective observational cohort study, it represents the experience of only a single center. That the sample was relatively small may have led to discrepancies in some observations. Although all participants received guideline-based medical therapy and lifestyle changes after stent implantation, we not assess long-term treatment and lifestyle changes; these factors may be confounds in ISR development. In addition, the 12-month follow-up was relatively short, and use of different imaging modalities was lacking. Therefore, multicenter, prospective, and larger-sample studies should be conducted to elucidate the exact role of ET-1 in predicting ISR occurrence after vertebral artery stenting.

## Conclusions

ISR after VAO stent implantation is common in clinical practice, and our study found that serum ET-1 can be used as an independent predictor. Future studies with larger samples will be needed to confirm these findings.

## Data Availability

The data that support the findings of this study are available from the corresponding author upon reasonable request.
